# The Effect of Preoperative Stoma Education Including a Wearable Dummy Stoma on Postoperative Stoma–Related Anxiety, Adjustment and Self‐Efficacy: a Randomised Controlled Trial

**DOI:** 10.1111/ijn.70121

**Published:** 2026-03-10

**Authors:** Hacer Akbayrak, Yasemin Uslu

**Affiliations:** ^1^ Surgical Department, Acıbadem Altunizade Hospital Acıbadem Health Group Istanbul Turkey; ^2^ Istanbul University Faculty of Nursing Istanbul Turkey

**Keywords:** anxiety, nursing, patient education, stoma care, stoma self‐efficacy

## Abstract

**Aims:**

This study aimed to determine the effects of preoperative stoma education including a wearable dummy stoma on stoma‐related anxiety, adjustment and self‐efficacy in patients undergoing colostomy/ileostomy.

**Materials and Methods:**

A randomised controlled trial was conducted in Istanbul between 2021 and 2023. The study included 78 patients aged 18 years or older who underwent colostomy/ileostomy for any indication. Patients in the experimental group received structured preoperative stoma education including a wearable dummy stoma designed as an artefact‐based tool to support hands‐on stoma care training, whereas those in the control group received standard care. Data were collected with the Stoma Self‐Efficacy Scale, Ostomy Adjustment Inventory (at discharge and at weeks 3 and 6) and State Anxiety Inventory (during the first stoma encounter).

**Results:**

Compared with those in the control group, patients in the experimental group demonstrated significantly lower anxiety levels during the first postoperative stoma encounter (*p* < 0.001), better stoma adjustment (*p* < 0.001 at discharge and at weeks 3 and 6) and greater stoma self‐efficacy levels (*p* < 0.001 at discharge and at weeks 3 and 6).

**Conclusion:**

Preoperative stoma education including a wearable dummy stoma effectively reduced stoma‐related anxiety during the first stoma encounter and improved stoma adjustment and self‐efficacy in patients.

**Trial Registration:**

NCT05308693.

## Summary

**What Is Already Known About This Topic?:**

- Preoperative education enhances self‐efficacy, yet postoperative pain and cognitive overload often hinder the acquisition of practical stoma care skills.
- While digital tools are increasingly utilised, evidence remains limited regarding tangible, artefact‐based materials that address the sensory‐tactile demands of stoma management.

**What This Paper Adds?:**

- Wearable dummy stoma significantly reduces stoma‐related anxiety during the first postoperative stoma encounter.
- Patients who received preoperative education via wearable dummy stoma demonstrated better stoma adjustment and higher self‐efficacy at discharge and throughout the 6‐week follow‐up.
- Wearable dummy stoma provides an interactive, experience‐based learning approach that enhances patient engagement and knowledge retention.

**The Implications of This Paper:**

- The incorporation of wearable dummy stoma into preoperative education enhances psychomotor mastery and patient readiness for postoperative self‐care.
- Utilising wearable dummy stoma provides a cost‐effective, experiential learning strategy to foster sustained self‐efficacy and facilitate long‐term psychosocial adjustment to a stoma.

**Abbreviations**
IBD: inflammatory bowel disease
SD: standard deviation

## 1 Introduction

The increasing incidence of colorectal cancer and advances in surgical techniques are leading to a greater number of people living with a stoma (Kugler et al. 2021; Rutherford et al. 2020). Consequently, stoma patients represent a patient population with significant unmet needs in structured patient education and ongoing support for stoma self‐care and adaptation (Rosenberg and McGee 2023). To address these needs, patients must acquire the necessary knowledge and skills to adapt to their stoma and develop self‐efficacy in stoma care (Kozell et al. 2014). Self‐efficacy refers to individuals' beliefs in their performance abilities, which influence their motivation, behaviour and responses to challenges (Bandura 1995). Higher self‐efficacy levels are associated with greater stoma adjustment (Özden and Kılıç 2023). Patient education and self‐care practices are essential for promoting stoma adaptation and stoma‐related self‐efficacy, both of which have been shown to improve quality of life (Iovino et al. 2023; Marcomini et al. 2024; Rosenberg and McGee 2023). Individuals with a stoma require training and counselling both before and after surgery to effectively manage and adapt to life with a stoma. Patient education focuses on various aspects, including stoma self‐care skills, diet, physical activity, travel, sexual activity and adaptation to a new body image (Pate et al. 2022; Rosenberg and McGee 2023). In the early postoperative period, patients and their relatives may not be able to concentrate on education and acquire self‐care skills because of postoperative pain, physical discomfort, fatigue, cognitive overload and heightened anxiety or depressive symptoms (Iovino et al. 2023). Therefore, stoma education should be started during the preoperative period (Rosenberg and McGee 2023). Preoperative education prevents stoma‐related complications, shortens hospital stays, improves patient competence and reduces patients' anxiety levels (Harris et al. 2020; Pouresmail et al. 2019). Before discharge, individuals with a stoma should be able to perform essential stoma self‐care tasks, including pouch management and peristomal skin care and demonstrate the knowledge and psychomotor skills required to manage their stoma safely and effectively (Panattoni et al. 2023). With modern surgical methods, patients have shorter hospital stays, thus shortening the allocated educational period. Owing to the limited time in the hospital, it is necessary to provide effective education to patients in a short time (Millard et al. 2020).

Despite the increasing use of modern approaches such as multimedia‐supported teaching, mobile applications and telehealth‐based methods (Iovino et al. 2024; Liu et al. 2023; Özkaya and Harputlu 2024; Uslu and Seyhan Ak 2024; Wang et al. 2021; Yiğitoğlu and Şendir 2021), comparatively limited attention has been given to tangible, artefact‐based materials that facilitate tactile learning. As defined in Vygotsky's (1978) sociocultural theory, artefacts are human‐made tools that mediate the relationship between the learner and the object, transforming mental functioning (Vygotsky 1978). In nursing, these artefacts bridge the gap between theory and clinical practice (Pouresmail et al. 2019; Stoffels et al. 2024).

This study focuses on a wearable dummy stoma as an artefact‐based tool. Grounded in Kolb's experiential learning theory, which emphasises learning through concrete experience and active participation (Kolb 2014), these wearable artefacts enable patients to engage actively in tasks, supporting the development of psychomotor skills and self‐efficacy (Nurunnabi et al. 2022). The aim of this study was to evaluate the effect of the use of wearable dummy stoma in preoperative stoma education on patients' stoma‐related anxiety during their initial encounter with the stoma, as well as their postoperative adjustment and self‐efficacy.

## 2 Methods

### 2.1 Study Design

This study was a randomised controlled trial and was registered at ClinicalTrials.gov (NCT05308693). The study was organised according to the CONSORT (Consolidated Standards of Reporting Trails) guidelines (Boutron et al. 2017).

### 2.2 Hypotheses of the Study

The study hypotheses were as follows:

*The use of wearable dummy in stoma education affects patients' anxiety during their first stoma encounter*.

*The use of wearable dummy in stoma education affects patients' stoma self‐efficacy*.

*The use of wearable dummy in stoma education affects patients' stoma adjustment*.

### 2.3 Population and Sample

The study was conducted in the surgical clinics of two hospitals in Istanbul between October 2021 and April 2023. Patients over 18 years of age who underwent colostomy/ileostomy for any reason (e.g., cancer, ulcerative colitis, Crohn's disease, hernia) were included in the study. Patients who were diagnosed by a doctor with any psychiatric or neurological illnesses (e.g., dementia, depression), visual or hearing impairments, who underwent postoperative revision surgery or stoma closure during the data collection process, who experienced postoperative complications or who required prolonged hospital stays were excluded from the study.

The participants were allocated to study groups via a random sampling method with an equal distribution ratio (distribution ratio = 1:1). Randomisation was performed via an online randomiser (https://www.randomlists.com/random‐letters).

The sample size was determined a priori, prior to participant recruitment, by performing a power analysis via GPower version 3.1.9.4. On the basis of the reference study by Pouresmail et al. (2019), a minimum of 78 participants were required to achieve 80% power (1 − *β*) at a significance level of *α* = 0.05, assuming a moderate effect size (Cohen's *d* = 0.65) (Pouresmail et al. 2019).

Although 78 patients were originally enrolled, eight patients were excluded during the data collection phase, and the study was completed with 70 patients. A post hoc power analysis was conducted to assess the adequacy of the sample. To evaluate the adequacy of the final sample size following participant attrition, a post hoc power analysis was conducted. Using the lowest observed effect size for stoma adjustment between the control group and the experimental group (mean difference = 11.97, SD = ±11.56), the calculated effect size was *d* = 1.09. On the basis of this effect size, a post hoc power analysis via GPower indicated a test power of 97.9% at the 95% confidence level (1 − *α*). On the basis of these findings, the research sample was sufficient; therefore, no additional patients were recruited.

### 2.4 Stoma Education Procedures

In‐hospital stoma care education was provided by two ostomy nurses. Both hospitals had similar characteristics and practice procedures. Prior to the study, the ostomy nurses received approximately 1 h of training on the use of the wearable dummy stoma. To ensure intervention fidelity, pilot sessions were conducted with two patients, during which the nurses' instructional techniques were observed and standardised. For both the experimental and control groups, education was delivered face‐to‐face in the patient's room 1 day prior to surgery, ensuring completion at least 6 h before the operative procedure.

#### 2.4.1 Control Group (Standard Stoma Education)

The control group received routine preoperative education via institutional brochures. The curriculum covered digestive anatomy, stoma classification and self‐management principles such as skin care, hygiene and nutrition. Instructions for emptying and changing the pouch system were provided through verbal explanations and visual aids. To ensure temporal parity with the experimental group, each session lasted approximately 30–45 min, representing the total direct nursing time dedicated to preoperative preparation. During this session, patients were encouraged to ask questions, and nurses provided clarification as needed. No additional structured follow‐up education sessions were scheduled as part of standard care.

#### 2.4.2 Experimental Group (Stoma Education Including a Wearable Dummy Stoma)

**FIGURE 1:**
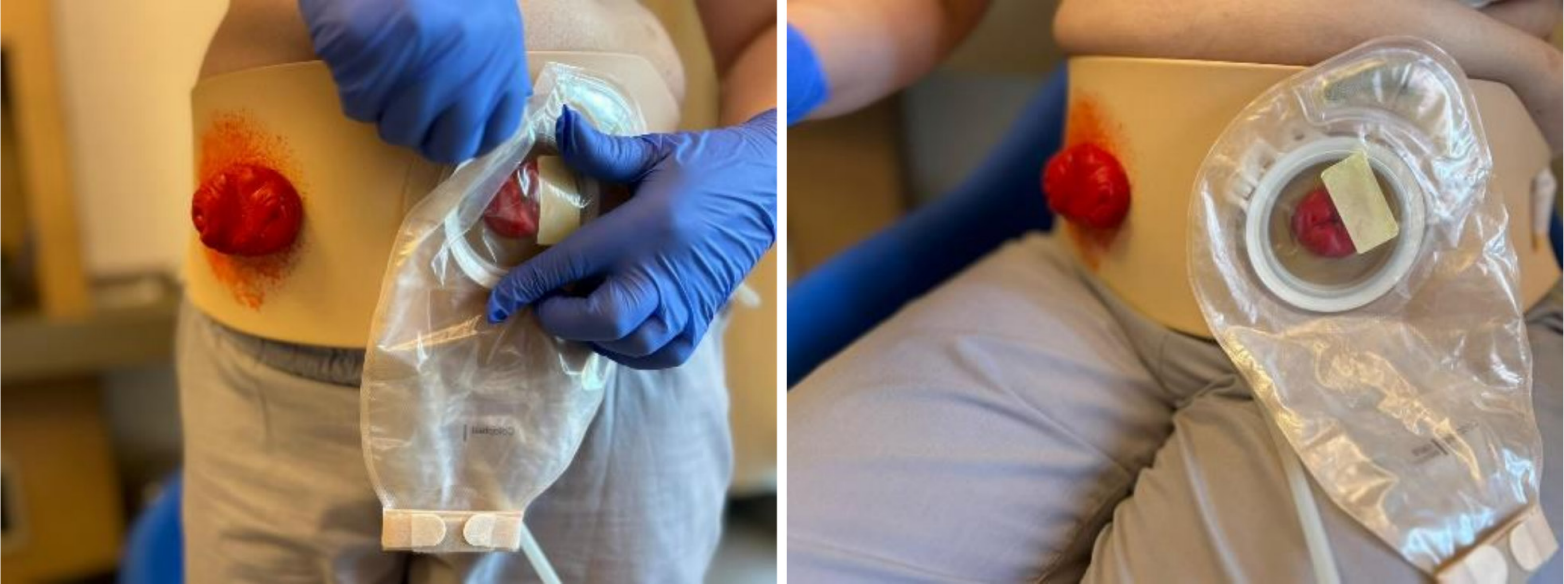
Wearable dummy stoma on a patient standing and sitting.

The experimental group received preoperative education via wearable dummy stoma. This artefact‐based tool is secured to the patient's abdomen via hook‐and‐loop fasteners and is adjustable to four anatomical positions (right/left, lower/upper quadrants). It features interchangeable modules for urostomy, colostomy and ileostomy, each capable of simulating realistic effluent (urine or stool) output. The material is washable, reusable and designed to facilitate the application of various stoma care accessories, including powders, pastes and belts. This wearable design allows patients to visualise the stoma's location on their own body and observe effluent passage according to the specific stoma type (Figure 1).

The intervention followed a standardised protocol consisting of a single 30–45 min preoperative session. Initially, patients reviewed various stoma products and practiced basic pouching techniques on a flat surface. The wearable dummy was subsequently secured to the patient's abdomen to facilitate supervised, hands‐on practice. The participants performed complex psychomotor tasks, including the application and removal of adapters with and without paste and the management of different pouch systems. While the session duration was adapted to individual patient engagement and inquiries, every participant was required to complete at least one full cycle of stoma pouch application and removal to ensure procedural competence.

### 2.5 Data Collection Tools

**FIGURE 2:**
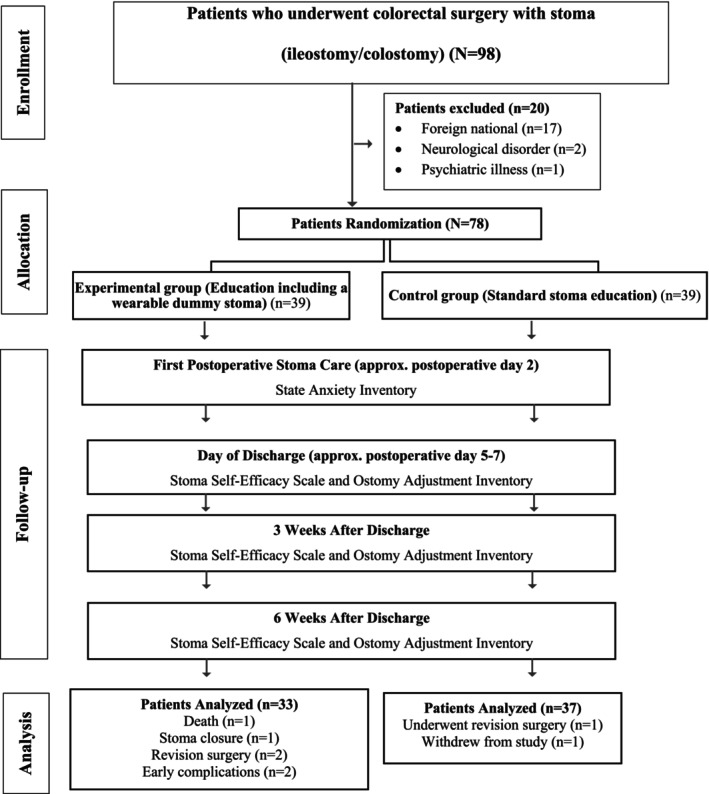
CONSORT research flow diagram.

Data were collected with the Patient Identification Form, the Stoma Self‐Efficacy Scale (SSES), Ostomy Adjustment Inventory (OAI) and State–Trait Anxiety Inventory (STAI). The SSES and OAI were administered three times: at discharge and at 3 and 6 weeks after discharge. The STAI was administered on the second postoperative day, at the time of first stoma care. The study design is shown in Figure 2.

#### 2.5.1 Patient Identification Form

The form, prepared by the researchers, collected data on patients' sociodemographic characteristics (e.g., sex, age, education level) and surgery‐related information (e.g., type of surgery, diagnosis). Some questions were directed to the patient, whereas others were obtained from the patient's records or the healthcare team.

#### 2.5.2 SSES

The SSES was developed by Bekkers to assess stoma‐related self‐efficacy levels in individuals with stomas (Jin et al. 2020). Karaçay et al. (2020) conducted Turkish adaptation and validity and reliability studies of the tool. The SSES consists of 22 items rated on a 5‐point Likert‐type scale. It has two subscales: stoma care self‐efficacy and social self‐efficacy. Scores range from 22 to 110, with higher scores indicating a better level of self‐efficacy. In the Turkish validity–reliability study of the scale, Cronbach's *α* value was found to be 0.95 (Karaçay et al. 2020). In this study, Cronbach's alpha (*α*) coefficient values were determined to be between 0.865 and 0.951.

#### 2.5.3 OAI‐23

The OAI was developed by Simmons, Smith and Maekawa to evaluate individuals' adjustment to living with a stoma (Simmons et al. 2007). It was adapted to Turkish by Karadag et al. (2011). The OAI consists of 23 items rated on a 5‐point Likert‐type scale and is organised into four subscales: anger, social engagement, anxiety and acceptance. Scores range between 0 and 92. A higher score indicates better adjustment to the stoma. In the Turkish validity–reliability study of the scale, the Cronbach's *α* value was found to be 0.874 (Karadag et al. 2011). In this study, Cronbach's alpha (*α*) coefficient values were determined to be between 0.808 and 0.684.

#### 2.5.4 State Anxiety Inventory

This anxiety inventory was developed by Spielberg et al. in 1970 and adapted to Turkish by Öner and Le Compte in 1977. The items utilise a 4‐point Likert‐type scale, and the total score ranges from 20 to 80. A high scale score is directly proportional to a high anxiety level. The state anxiety scale reflects the evaluation of how the patient feels at the present moment. The higher the score is, the higher the anxiety level (Ebrinc 2000). After surgery, patients see their stoma for the first time on postoperative day 2. First, the stoma bag and adapter were not removed due to surgical healing. Therefore, the anxiety scale was used during this period. In this study, Cronbach's alpha (*α*) coefficient values were determined to be between 0.881 and 0.899.

### 2.6 Data Analysis

The SPSS (Statistical Package for the Social Sciences) version 27 software (IBM Corp., Armonk, NY, United States) was used for the statistical analyses. Skewness and kurtosis coefficients were calculated to determine whether the scores obtained from each continuous variable were normally distributed. The calculated values ranged between −1.5 and +1.5. For continuous variables, an independent sample *t* test was used for comparisons between variables. Repeated‐measures ANOVA was used for within‐group comparisons. The Bonferroni correction was used to determine which measurement caused the statistically significant difference. Chi‐square tests (Pearson's chi‐square test, Yates' corrected chi‐square test and Fisher's exact test) were used for qualitative comparisons between groups. One‐way multivariate analysis of variance (MANOVA) was conducted to determine whether the OAI, SSES and STAI‐I scores differed significantly according to the experimental group. The scale's reliability was evaluated via Cronbach's alpha analysis. The results were evaluated within 95% confidence intervals, and significance was accepted at *p* < 0.05.

### 2.7 Ethical Aspects of the Study

Ethical approval was obtained from the Acıbadem University Medical Research Ethics Committee (2021‐13/04), and written and verbal informed consent was obtained from all patients prior to data collection.

## 3 Results

### 3.1 Participant Characteristics

The study included a total of 70 patients, with 37 patients allocated to the control group and 33 to the experimental group. The mean age of the patients was 53.51 ± 16.03 years; 50% were male, 72.9% were married, 61.4% were actively employed and 92.9% had a chronic disease (Table 1). The most common diagnosis was colorectal cancer (64.3%), the surgeries were most frequently open (38.6%) or laparoscopic (38.6%), and 82.9% of the patients had a temporary stoma. There was no difference in demographic or surgical characteristics between the control and experimental groups (Table 1) (*p* > 0.05).

**TABLE 1:** Characteristics of patients who underwent stoma surgery.

	Total (*n* = 70)	Experimental group (*n* = 33)	Control group (*n* = 37)	Test value	*p*
	*n* (%)	*n* (%)	*n* (%)		
Age (years), mean ± SD	53.51 ± 16.03	55.15 ± 17.00	52.05 ± 15.19	0.805^a^	0.424
Gender				2.064^c^	0.151
Male	35 (50.0)	13 (39.4)	22 (59.5)		
Famale	35 (50.0)	20 (60.6)	15 (40.5)		
Marital status				0.061^c^	0.806
Married	51 (72.9)	25 (75.8)	26 (70.3)		
Single	19 (27.1)	8 (24.2)	11 (29.7)		
Education				2.638^b^	0.267
Primary education	14 (20.0)	6 (18.2)	8 (21.6)		
High school	33 (47.1)	13 (39.4)	20 (54.1)		
University	23 (32.9)	14 (42.4)	9 (24.3)		
Employment status				0.144^c^	0.704
Employed	43 (61.4)	19 (57.6)	24 (64.9)		
Not employed	27 (38.6)	14 (42.4)	13 (35.1)		
Chronic disease				‐^d^	0.999
Yes	65 (92.9)	31 (93.9)	34 (91.9)		
No	5 (7.1)	2 (6.1)	3 (8.1)		
Type of chronic disease					
Coronary artery disease	29 (42.0)	15 (46.9)	14 (37.8)	0.264^c^	0.607
Hypertension and/or diabetes	24 (34.3)	12 (36.4)	12 (32.4)	0.009^c^	0.925
Respiratory diseases	4 (5.7)	2 (6.1)	2 (5.4)	‐^d^	0.999
Type of surgery				1.285^b^	0.526
Open	27 (38.6)	11 (33.3)	16 (43.2)		
Robotic	16 (22.9)	7 (21.2)	9 (24.3)		
Laparoscopic	27 (38.6)	15 (45.5)	12 (32.4)		
Diagnosis				1.305^c^	0.253
Malignancy (colorectal cancer)	45 (64.3)	24 (72.7)	21 (56.8)		
Other (IBD/obstruction)	25 (35.7)	9 (27.3)	16 (43.2)		
Ostomy				1.371^c^	0.242
Permanent	12 (17.1)	8 (24.2)	4 (10.8)		
Temporary	58 (82.9)	25 (75.8)	33 (89.2)		
Person performing stoma care				5.462^b^	0.526
Self	34 (48.6)	16 (48.5)	18 (48.6)		
Spouse	18 (25.7)	5 (15.2)	13 (35.1)		
Child	12 (17.1)	8 (24.2)	4 (10.8)		
Caretaker	6 (8.6)	4 (12.1)	2 (5.4)		
Previous experience with stoma				‐^d^	0.999
Yes	1 (1.4)	0 (0)	1 (2.7)		
No	69 (98.6)	33 (100)	36 (97.3)		

*Note:* All comparisons: *p* > 0.05. a: Independent samples *t* test, b: Pearson's chi‐square test, c: Yates corrected chi‐square test, d: Fisher's exact test.

### 3.2 Effects of the Intervention on Initial Stoma‐Related Anxiety (H1)

State anxiety levels during the first postoperative stoma care (approximately postoperative day 2) were significantly lower in patients in the experimental group than in those in the control group (*Z* = −5.380; *p* < 0.001) (Table 2).

**TABLE 2:** Stoma‐related state anxiety, adjustment and self‐efficacy score distributions of patients undergoing stoma surgery.

	Experimental group (*n* = 33)	Control group (*n* = 37)	Test value^a^	*p*
	Mean ± SD	Mean ± SD		
State Anxiety Inventory	36.24 ± 8.15	48.16 ± 10.14	t = 5.380	< 0.001*
Stoma Self‐Efficacy Scale				
At discharge^1^	63.36 ± 12.72	49.92 ± 18.77	3.540	< 0.001*
3 weeks^2^	78.39 ± 10.51	59.08 ± 17.07	5.767	< 0.001*
6 weeks^3^	84.73 ± 10.86	68.24 ± 17.68	4.753	< 0.001*
Test value^b^	43.603	22.153		
p value	< 0.001*	< 0.001*		
Post hoc differenceᶜ	1 < 2 < 3	1 < 2 < 3		
Ostomy Adjustment Inventory				
At discharge^1^	54.67 ± 9.78	42.70 ± 11.56	4.644	< 0.001*
3 weeks^2^	64.72 ± 11.13	49.57 ± 12.10	5.382	< 0.001*
6 weeks^3^	68.42 ± 13.20	44.19 ± 12.04	8.035	< 0.001*
Test value^b^	F = 18.853	5.677		
p value	< 0.001*	0.005*		
Post hoc differenceᶜ	1 < 2, 3	1 < 2		

*Note:* a: Independent sample *t* test, b: repeated‐measures ANOVA, c: post hoc pairwise comparisons were performed via the Bonferroni correction. The Bonferroni‐adjusted significance threshold was set at *p* < 0.017 (0.05/3).

SD: standard deviation.

* *p* < 0.05.

### 3.3 Effects of the Intervention on Stoma Self‐Efficacy (H2)

SSES scores were also significantly higher (higher self‐efficacy) in the experimental group at all measurement points, including at discharge (*Z* = 3.540; *p* < 0.001), at 3 weeks (*Z* = 5.767; *p* < 0.001) and at 6 weeks (*Z* = 4.753; *p* < 0.001) (Table 2). Both groups showed a statistically significant increase in the SSES score at each time point (experimental group, *χ* ^2^ = 43 603; *p* < 0,001; control group, *χ* ^2^ = 22 153; *p* < 0,001) (Table 2).

### 3.4 Effects of the Intervention on Stoma Adjustment (H3)

The mean OAI score was significantly greater (better adjustment) in the experimental group at all measurement points, including at discharge (*Z* = 4.644; *p* < 0.001), at 3 weeks (*Z* = 5.382; *p* < 0.001) and at 6 weeks (*Z* = 8.035; *p* < 0.001). A significant change in the OAI score was observed within each group (experimental group, *χ* ^2^ = 18 853; *p* < 0,001; control group, *χ* ^2^ = 5677; *p* = 0,005), but the score increased steadily between each time point only in the experimental group (Table 2).

### 3.5 Multivariate Analysis of Intervention Outcomes

The results of the one‐way MANOVA test indicated a statistically significant difference in patients' OAI [*λ* = 0.448; *F* = 20.976 to 61.781; *p* < 0.001], SSES [*λ* = 0.448; *F* = 11.829 to 30.844; *p* < 0.001 and *p* < 0.01] and STAI‐I [*λ* = 0.448; *F* = 27.628; *p* < 0.001] scores across all measurement points across all the experimental groups. An examination of the highest *η* ^2^ values revealed that the educational intervention accounted for 48% of the variance in the OAI score at Week 6 (*η* ^2^ = 0.48) and 31.5% of the variance in the SSES score at Week 3 (*η* ^2^ = 0.315) (Table 3).

**TABLE 3:** MANOVA test results for the experimental group.

Effect	Dependent variable	Wilks Lambda (*λ*)	*F*(7–61)	*p*	*η* ^2^(eta)
Experimental group	OAI‐At discharge		20.976	< 0.001	0.238
	OAI‐3 weeks		28.965	< 0.001	0.302
	OAI‐6 weeks	0.448	61.781	< 0.001	0.480
	SSES‐At discharge		11.829	0.001	0.150
	SSES‐3 weeks		30.844	< 0.001	0.315
	SSES‐6 weeks		20.473	< 0.001	0.234
	STAI‐I		27.628	< 0.001	0.292

## 4 Discussion

The findings of the present study indicate that patients who received preoperative stoma education including a wearable dummy stoma experienced significantly lower levels of stoma‐related anxiety during their initial encounter. Additionally, these patients exhibited higher levels of stoma self‐efficacy and adjustment following discharge than did those in the standard education group.

Preoperative stoma education has been shown to significantly reduce postoperative anxiety in patients undergoing ostomy surgery (Harris et al. 2020; Ruiz Hernández et al. 2021). In this study, the wearable dummy stoma enabled patients to experience the stoma on their own bodies prior to surgery. Owing to the high realism level of the material, patients could mentally and emotionally prepare themselves for life with a stoma. Consequently, they experienced lower anxiety when encountering their real stoma postoperatively. This finding is particularly noteworthy, as there is currently no research exploring the impact of different stoma education methods on patients' emotional responses to their first stoma encounter. Our hypothesis (H1) was confirmed, as the use of the wearable dummy in stoma education significantly reduces patients' anxiety during their first stoma encounter.

Self‐efficacy, defined as an individual's belief in their ability to perform specific behaviours, is a crucial determinant in the adoption and maintenance of positive health behaviours (Bandura 1995; Su et al. 2016). Previous studies have demonstrated that various educational interventions, such as multimedia programs, phone follow‐ups and mobile applications, increase stoma‐related self‐efficacy (Bozkul et al. 2024; Nasiriziba et al. 2020; Pouresmail et al. 2019; Song et al. 2021; Yiğitoğlu and Şendir 2021). Our results suggest that wearable dummy stoma provides a distinct advantage by enabling active, hands‐on engagement. Consistent with experiential learning principles, repeated practice and direct interaction with the learning task may have supported patients' confidence and preparedness for stoma self‐care (Shorey et al. 2021). Our hypothesis (H2) was confirmed, as the use of wearable dummy in stoma education positively affects patients' stoma self‐efficacy.

Studies have shown that higher stoma‐related self‐efficacy levels are associated with better adjustment (Jin et al. 2020; Özden and Kılıç 2023; Pouresmail et al. 2019). Jin et al. (2020) reported that, over time, patients became increasingly adept at managing their stoma independently (Jin et al. 2020). While standard education also contributed to patient adjustment, its impact was less pronounced than that of wearable dummy stoma. These findings suggest that tangible, patient‐centred methods facilitate more effective information processing and adaptation than do conventional brochures (Goodman et al. 2022). Our hypothesis (H3) was confirmed, as the use of wearable dummy in stoma education positively affects patients' adjustment to live with a stoma.

Multivariate analysis further demonstrated that wearable dummy stoma was associated with meaningful longitudinal improvements in all outcomes. The large effect sizes, particularly for adjustment at 6 weeks and self‐efficacy at 3 weeks, underscore the clinical relevance of this approach. These findings support experiential learning theories that emphasise active participation and skill rehearsal in fostering self‐care mastery.

### 4.1 Study Limitations

The study was limited to adult patients who underwent stoma surgery in the two hospitals where the study was conducted. Patient outcomes were monitored for only 6 weeks. It is not known how patient outcomes are affected over the subsequent longer term. The results were based on patients' self‐reports, which may be subject to various forms of bias. In addition, although the duration of the preoperative education sessions was standardised for both groups, minor variations in the amount of time and individual attention provided by the nurses may have occurred due to patient engagement and questions. This variability may have influenced patient outcomes and should be considered when interpreting the results. Research results cannot be generalised to all stoma patients.

## 5 Conclusion

This study demonstrated that patients who received preoperative stoma education including wearable dummy experienced lower stoma‐related anxiety, greater self‐efficacy and better adjustment than did those who received standard education. These positive effects persisted and continued to improve over the 6‐week follow‐up period. Wearable dummy stoma, by promoting active patient participation and experiential learning, significantly enhances patient outcomes. Given their demonstrated effectiveness, such materials may be considered for integration into standard preoperative stoma education programs as a structured, hands‐on component to support both psychological adaptation and practical skill development.

## Author Contribution

H.A. and Y.U. conceived the study and determined the methodology. H.A. collected the data. All authors contributed to the planning of the research, analysis, preparation, review and finalisation of the article. All authors reviewed the final manuscript before submitting for publication.

## Conflicts of Interest

The authors declare no conflicts of interest.

## Data Availability

The data that support the findings of this study are available from the corresponding author upon reasonable request.

## References

[ijn70121-bib-0001] Bandura, A. 1995. “Exercise of Personal and Collective Efficacy in Changing Societies.” In Self‐efficacy in changing societies, 1–45. Cambridge University Press.

[ijn70121-bib-0002] Boutron, I. , D. G. Altman , D. Moher , K. F. Schulz , and P. Ravaud . 2017. “CONSORT Statement for Randomized Trials of Nonpharmacologic Treatments: A 2017 Update and a CONSORT Extension for Nonpharmacologic Trial Abstracts.” Annals of Internal Medicine 167, no. 1: 40–47. 10.7326/M17-0046. 28630973

[ijn70121-bib-0003] Bozkul, G. , S. Senol Celik , and H. Nur Arslan . 2024. “Nursing Interventions for the Self‐Efficacy of Ostomy Patients: a Systematic Review.” Journal of Tissue Viability 33, no. 2: 165–173. 10.1016/j.jtv.2024.04.006. 38627154

[ijn70121-bib-0004] Ebrinc, S. 2000. “Psychiatric Rating Scales and Their Use in Clinical Studies.” Bulletin of Clinical Psychopharmacology 10, no. 2: 109–116.

[ijn70121-bib-0005] Goodman, W. , M. Allsop , A. Downing , et al. 2022. “A Systematic Review and meta‐Analysis of the Effectiveness of Self‐Management Interventions in People With a Stoma.” Journal of Advanced Nursing 78, no. 3: 722–738. 10.1111/jan.15085. 34708416

[ijn70121-bib-0006] Harris, M. S. , K. Kelly , and C. Parise . 2020. “Does Preoperative Ostomy Education Decrease Anxiety in the New Ostomy Patient? A Quantitative Comparison Cohort Study.” Journal of Wound, Ostomy, and Continence Nursing 47, no. 2: 137–139. 10.1097/WON.0000000000000623. 32150139

[ijn70121-bib-0007] Iovino, P. , M. De Maria , F. Corvese , et al. 2023. “The Influence of Patient and Caregiver Depression on Patient Self‐Care and Caregiver Contribution to Self‐Care in Ostomy: a Dyadic Analysis.” Journal of Clinical Nursing 32, no. 17–18: 6441–6449. 10.1111/jocn.16676. 36880219

[ijn70121-bib-0008] Iovino, P. , E. Vellone , A. Campoli , et al. 2024. “Telehealth vs in‐Person Education for Enhancing Self‐Care of Ostomy Patients (Self‐Stoma): Protocol for a Noninferiority, Randomized, Open‐Label, Controlled Trial.” PLoS ONE 19, no. 6: e0303015. 10.1371/journal.pone.0303015. 38924038 PMC11206953

[ijn70121-bib-0009] Jin, Y. , H. Ma , and M. Jiménez‐Herrera . 2020. “Self‐Disgust and Stigma Both Mediate the Relationship Between Stoma Acceptance and Stoma Care Self‐Efficacy.” Journal of Advanced Nursing 76, no. 10: 2547–2558. 10.1111/jan.14457. 32700799

[ijn70121-bib-0010] Karaçay, P. , E. Toğluk Yigitoglu , and A. Karadağ . 2020. “The Validity and Reliability of the Stoma Self‐Efficacy Scale: a Methodological Study.” International Journal of Nursing Practice 26, no. 6: e12840. 10.1111/ijn.12840. 32301580

[ijn70121-bib-0011] Karadag, A. , Z. Baykara , H. Korkut , and B. Cßelik . 2011. “Adaptation of the Ostomy Adjustment Inventory Into Turkish Language.” Turkish Journal of Surgery 27: 206–211.

[ijn70121-bib-0012] Kolb, D. A. 2014. Experiential Learning: Experience as the Source of Learning and Development. FT press.

[ijn70121-bib-0013] Kozell, K. , M. Frecea , and J. T. Thomas . 2014. “Preoperative Ostomy Education and Stoma Site Marking.” Journal of Wound, Ostomy, and Continence Nursing 41, no. 3: 206–207. 10.1097/won.0000000000000031. 24805169

[ijn70121-bib-0014] Kugler, C. M. , J. Breuing , T. Rombey , et al. 2021. “The Effect of Preoperative Stoma Site Marking on Risk of Stoma‐Related Complications in Patients With Intestinal Ostomy—Protocol of a Systematic Review and Meta‐Analysis.” Systematic Reviews 10, no. 1: 146. 10.1186/s13643-021-01684-8. 33980317 PMC8117581

[ijn70121-bib-0015] Liu, Y. , L. Wang , and L. Zhu . 2023. “The Impact of Stoma Management Education on the Self‐Care Abilities of Individuals With an Intestinal Stoma.” Gastrointestinal Nursing 21, no. Sup4: S14–S21. 10.12968/bjon.2023.32.6.S28 36952366

[ijn70121-bib-0016] Marcomini, I. , P. Iovino , L. Rasero , D. F. Manara , E. Vellone , and G. Villa . 2024. “Self‐Care and Quality of Life of Ostomy Patients: a Structural Equation Modeling Analysis.” Nursing Reports 14, no. 4: 3417–3426. 10.3390/nursrep14040247. 39585138 PMC11587398

[ijn70121-bib-0017] Millard, R. , D. Cooper , and M. J. Boyle . 2020. “Improving Self‐Care Outcomes in Ostomy Patients via Education and Standardized Discharge Criteria.” Home Healthc Now 38, no. 1: 16–23. 10.1097/nhh.0000000000000816. 31895893

[ijn70121-bib-0018] Nasiriziba, F. , M. Saati , and H. Haghani . 2020. “Correlation Between Self‐Efficacy and Self‐Esteem in Patients With an Intestinal Stoma.” British Journal of Nursing 29, no. 16: S22–s29. 10.12968/bjon.2020.29.16.S22. 32901542

[ijn70121-bib-0019] Nurunnabi, A. S. M. , R. Rahim , D. Alo , et al. 2022. “Experiential Learning in Clinical Education Guided by the Kolb's Experiential Learning Theory.” International Journal of Human Health Sciences 6, no. 2: 155–160. 10.31344/ijhhs.v6i2.438.

[ijn70121-bib-0020] Özden, Z. M. , and M. Kılıç . 2023. “The Effect of Self‐Efficacy Levels of Patients With Intestinal Stoma on Stoma Adaptation.” Supportive Care in Cancer 31, no. 5: 252. 10.1007/s00520-023-07702-w. 37036537 PMC10088733

[ijn70121-bib-0021] Özkaya, E. , and D. Harputlu . 2024. “The Effect of Education via Videoconferencing at Home on Individuals' Self‐Efficacy and Adaptation to Life With a Stoma: a Randomized Controlled Study.” Advances in Skin & Wound Care 37, no. 2: 86–94. 10.1097/ASW.0000000000000098. 38241451

[ijn70121-bib-0022] Panattoni, N. , R. Mariani , A. Spano , et al. 2023. “Nurse Specialist and Ostomy Patient: Competence and Skills in the Care Pathway. A Scoping Review.” Journal of Clinical Nursing 32, no. 17–18: 5959–5973. 10.1111/jocn.16722. 37073684

[ijn70121-bib-0023] Pate, K. , K. Powers , M. J. Coffman , and S. Morton . 2022. “Improving Self‐Efficacy of Patients With a New Ostomy With Written Education Materials: a Quality Improvement Project.” Journal of Perianesthesia Nursing 37, no. 5: 620–625. 10.1016/j.jopan.2021.11.020. 35260298

[ijn70121-bib-0024] Pouresmail, Z. , F. H. Nabavi , A. Abdollahi , M. T. Shakeri , and A. Saki . 2019. “Effect of Using a Simulation Device for Ostomy Self‐Care Teaching in Iran: a Pilot, Randomized Clinical Trial.” Wound Management & Prevention 65, no. 6: 30–39. 31373564

[ijn70121-bib-0025] Rosenberg, A. , and M. McGee . 2023. “Patient Education for Stoma Patients.” Seminars in Colon and Rectal Surgery 34, no. 2: 100952. 10.1016/j.scrs.2023.100952.

[ijn70121-bib-0026] Ruiz Hernández, C. , J. L. Gómez‐Urquiza , L. Pradas‐Hernández , et al. 2021. “Effectiveness of Nursing Interventions for Preoperative Anxiety in Adults: a Systematic Review With Meta‐Analysis.” Journal of Advanced Nursing 77, no. 8: 3274–3285. 10.1111/jan.14827. 33755246

[ijn70121-bib-0027] Rutherford, C. , F. Müller , N. Faiz , M. T. King , and K. White . 2020. “Patient‐Reported Outcomes and Experiences From the Perspective of Colorectal Cancer Survivors: Meta‐Synthesis of Qualitative Studies.” Journal of Patient‐Centered Outcomes 4, no. 1: 27. 10.1186/s41687-020-00195-9. PMC7183519 32335745

[ijn70121-bib-0028] Shorey, S. , and V. Lopez . 2021). Self‐Efficacy in a Nursing Context. In Haugan, G. E. M. , (Ed.), Health Promotion in Health Care—Vital Theories and Research (pp. 145–158). Springer. 10.1007/978-3-030-63135-2.

[ijn70121-bib-0029] Simmons, K. L. , J. A. Smith , K. A. Bobb , and L. L. Liles . 2007. “Adjustment to Colostomy: Stoma Acceptance, Stoma Care Self‐Efficacy and Interpersonal Relationships.” Journal of Advanced Nursing 60, no. 6: 627–635. 10.1111/j.1365-2648.2007.04446.x. 18039249

[ijn70121-bib-0030] Song, Q. F. , G. Yin , X. Guo , X. Lv , K. Yu , and C. Liu . 2021. “Effects of a Self‐Management Program for Patients With Colorectal Cancer and a Colostomy: a Nonrandomized Clinical Trial.” Journal of Wound, Ostomy, and Continence Nursing 48, no. 4: 311–317. 10.1097/won.0000000000000779. 34186549

[ijn70121-bib-0031] Stoffels, M. , L. A. Broeksma , M. Barry , et al. 2024. “Bridging School and Practice? Barriers to the Integration of 'Boundary Objects' for Learning and Assessment in Clinical Nursing Education.” Perspectives on Medical Education 13, no. 1: 392–405. 10.5334/pme.1103. 39006554 PMC11243767

[ijn70121-bib-0032] Su, X. , F. Qin , L. Zhen , et al. 2016. “Self‐Efficacy and Associated Factors in Patients With Temporary Ostomies.” Journal of Wound, Ostomy, and Continence Nursing 43, no. 6: 623–629. 10.1097/WON.0000000000000645. 27636323

[ijn70121-bib-0033] Uslu, Y. , and E. Seyhan Ak . 2024. “Current Approaches in Stoma Education.” Turkiye Klinikleri Surgical Nursing‐Special Topics 10, no. 1: 85–90.

[ijn70121-bib-0034] Vygotsky, L. S. 1978. Mind in Society: the Development of Higher Psychological Processes. Vol. 86. Harvard university press.

[ijn70121-bib-0035] Wang, S.‐Y. , T.‐H. Chang , and C.‐Y. Han . 2021. “Effectiveness of a Multimedia Patient Education Intervention on Improving Self‐Care Knowledge and Skills in Patients With Colorectal Cancer After Enterostomy Surgery: a Pilot Study.” Advances in Skin & Wound Care 34, no. 2: 1–6. 10.1097/01.ASW.0000725192.98920.c4. 33443916

[ijn70121-bib-0036] Yiğitoğlu, E. T. , and M. Şendir . 2021. “Effect of a Mobile Patient Education Application on Adjustment to Stoma and Development of Peristomal Skin Lesions: a Quasi‐Experimental Study.” Wound Management & Prevention 67, no. 12: 30–40. 35030542

